# Developing a holistic contingency plan: Challenges and dilemmas for cancer patients during the COVID‐19

**DOI:** 10.1002/cam4.3271

**Published:** 2020-07-20

**Authors:** Constantina Constantinou, Ourania Kolokotroni, Maria‐Cecilia Mosquera, Alexandros Heraclides, Christiana Demetriou, Peter Karayiannis, Annalisa Quattrocchi, Andreas Charalambous

**Affiliations:** ^1^ University of Nicosia Medical School Nicosia Cyprus

**Keywords:** cancer, contingency plan, COVID‐19, management, prevention

## Abstract

During the first quarter of 2020 the world is experiencing a pandemic of Severe Acute Respiratory Syndrome‑Coronavirus‑2 (SARS‑CoV‑2), a novel beta coronavirus that is responsible for the 2019 novel coronavirus disease (COVID‐19). The COVID‐19 pandemic revealed that healthcare systems around the world were not prepared to deal with either the direct effects of the pandemic or with the indirect effects that are imposed on the health of patients with chronic disorders such as cancer patients. Some challenges and dilemmas currently faced during the pandemic include the management of cancer patients during the treatment and follow‐up phases, the assessment of the safety of treatments currently used for the management of SARS‑CoV‑2 for use in cancer patients, the development of psychoeducation and emotional support for cancer patients and the safe conduct of clinical trials involving participation of cancer patients. Evidence from the literature supports the need for the urgent development of a holistic contingency plan which will include clear guidelines for the protection and comprehensive care of cancer patients. The implementation of such a plan is expected to have many beneficial effects by mainly minimizing the increased morbidity and mortality of cancer patients that could result as an adverse consequence of the COVID‐19 or future pandemics.

## Graphical abstract

The COVID‐19 pandemic has revealed that health care systems around the world were not prepared to deal with either the direct effects of the pandemic or with the indirect effects that are imposed on the health of cancer patients. The evidence from the literature supports that the development of a contingency plan is as urgent as ever to minimise the increased morbidity and mortality of cancer patients that could result as a consequence of the current COVID‐19 pandemic or any other future pandemics.

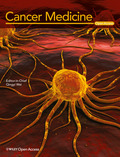

## 1 INTRODUCTION

During the first quarter of 2020 the world is experiencing a pandemic of Severe Acute Respiratory Syndrome‑Coronavirus‑2 (SARS‑CoV‑2), a novel beta coronavirus that is responsible for the 2019 novel coronavirus disease (COVID‐19).^1^, ^2^ It all started when at the end of December 2019, healthcare authorities reported unidentified cases of pneumonia in a seafood market in Wuhan, China. On the last day of January 2020, the World Health Organization (WHO) announced a Public Health Emergency of International Concern (PHEIC) and on 11th March 2020, the WHO formally declared the COVID‐19 outbreak a pandemic.^1^, ^3^ According to a WHO situation report through 29th April 2020, COVID‐19 was responsible for a total of 2 954 222 confirmed cases and 202,597 deaths.^4^

SARS‐CoV‐2 is an enveloped virus with a single‑stranded RNA genome of positive polarity.^5^, ^6^, ^7^ The most common symptoms are fever, dry cough, and fatigue. Some patients may present with headache and/or muscle pain but may have no upper respiratory symptoms.^8^, ^9^, ^10^, ^11^, ^12^ Men have a higher risk than women of being infected with the virus and patients with pre‐existing comorbidities such as diabetes, hypertension, and cardiovascular disease are among those at highest risk of not only acquiring the infection but also of having worse outcomes.^12^, ^13^ There is also concern that cancer patients may also be of higher risk which is exceedingly concerning from a public health perspective since cancer is amongst the most prevalent diseases worldwide.^14^

Cancer development is associated with a state of immunosuppression.^15^, ^16^ In addition, the management of the disorder (eg chemotherapy, immunotherapy, surgery, and hematopoietic transplantation) also leads to defects in the adaptive and innate immune systems.^16^, ^17^ For example, some conventional chemotherapies eliminate the function of T cells whereas some immunotherapeutic agents eliminate the function of B cells.^16^, ^17^ Therefore infection with SARS‑CoV‑2 can be a life‐threatening event for a cancer patient.^17^

Preliminary evidence in the literature supports that cancer patients infected with SARS‑CoV‑2 have a higher risk of disease severity and mortality compared to SARS‑CoV‑2 infected patients without cancer. A study by Liang et al,^18^ provided evidence that cancer patients (that had received chemotherapy or surgery within the past month) and cancer survivors (in routine follow‐up) who were infected with SARS‑CoV‑2 had a higher risk of severe events (defined as the percentage of patients being admitted to the intensive care unit requiring invasive ventilation, or death) (39%) compared to patients without cancer with SARS‑CoV‑2 (8%) adjusting for age and other known risk factors. In this study the mortality rate was significantly higher in cancer patients (5.6%) ^18^ compared to the virus’ overall case fatality proportion which appears to be about 3% in China. ^9^, ^19^Globally, about 3.4% of reported COVID‐19 cases died up to March 3rd, 2020.^20^ In another retrospective cohort study, the mortality of cancer patients infected with COVID‐19 was 26.9%^21^ whereas in COVID‐19 infected patients without cancer (of the same age group of 60‐69 years) the mortality was reported to be between 3.5%‐3.6% and 4.5%.^22^, ^23^ The most comprehensive data available to date is the Report of the WHO‐China Joint Mission on Coronavirus Disease published on February 28th, 2020.^24^ The report indicated that the case fatality rate for patients with cancer as a comorbid condition and laboratory confirmed infection with SARS‑CoV‑2 was 7.6% compared to patients with no comorbid condition and laboratory confirmed infection with SARS‑CoV‑2 which was 1.4%.^24^

An important factor associated with COVID‐19 disease severity and complications in patients with cancer and COVID‐19 is whether cancer patients are undergoing treatment at the time of infection. Zhang et al,^21^ reported that patients who had their last anti‐tumor treatment (including chemotherapy, immunotherapy, and radiation) within 14 days prior to infection with SARS‐CoV‐2 had a significantly increased risk of developing severe events (HR = 4.079, 95% CI 1.086‐15.322, *P* = .037).^21^ The results of another study found that cancer patients had an estimated twofold increased risk of COVID‐1 9 infection compared to the general population.^25^ Of the 1524 cancer patients examined, 12 were infected with SARS‐CoV‐2 and two of these patients died (16.7%), though one of the two patients died due to an unrelated cause. They also reported that 5 of the 12 patients were undergoing treatment at the time of contracting the virus, and it is possible that hospital visits for treatment were responsible for the increased incidence of COVID‐19 in cancer patients. Consequently, the researchers proposed that protocols should be developed to offer treatments to cancer patients in proper isolation to prevent the risk of SARS‐CoV‐2 infection.^25^

Many of the studies described are retrospective and the number of patients included in the studies was small.^26^ In addition, it has been proposed that smoking history and increased age may explain the increased susceptibility and severity of COVID‐19 in cancer patients compared to healthy individuals.^27^, ^28^, ^29^ In addition, the studies were carried out in China where the prevalence of specific cancers may be different than in the rest of the world.^26^ Furthermore, patients in China receive cancer therapy in hospitals, whereas in other countries treatment is provided in outpatient settings. Lastly, in these studies the cancer patients were treated in hospitals which were also COVID‐19 referral centers treating a high number of COVID‐19 patients, and this could lead to the cancer patients being exposed to a higher risk of infection with SARS‐CoV‐2 than if they had been treated in other healthcare centers.^26^

In the UK, NICE has developed the 'COVID‐19 rapid guideline: delivery of systematic anti‐cancer treatment', a generic guideline for all cancer patients.^31^ In the USA, guidelines have also been developed with regards to the management of cancer patients during the pandemic but these are specific to different types of cancers. Some examples include the guidelines developed by the American Society of Clinical Oncology (ASCO),^32^ the American Society of Breast Surgeons (ASBS),^33^ and the American College of Surgeons (ACS).^34^ However, currently, there are no international guidelines with regards to the management of all aspects of care of cancer patients during the COVID‐19 pandemic. Multiprofessional healthcare teams therefore need to be constantly evaluating the facts related to the COVID‐19 pandemic in order to make the most appropriate decisions for their cancer patients.^14^ Some of the questions addressed are the following: Should the cancer patients' management be continued or deferred and under which conditions? Should any specific measures be taken during treatment? Are there any specific measures to prevent infection? How will the pandemic affect ongoing or planned clinical trials, access to medication and hematopoietic cells.^14^ The purpose of the current review is to shed some light on the impact of the COVID‐19 pandemic on cancer patients and some challenges and dilemmas faced by the healthcare teams in their effort to continue to provide high quality healthcare to these patients.

## 2 MANAGING CANCER PATIENTS DURING THE TREATMENT AND FOLLOW‐UP PHASES

Cancer is a life‐threatening event and treatment should be offered at the appropriate periods and frequency as required by protocols. In addition, the provision of care to cancer patients requires good coordination between multiprofessional healthcare teams.^35^ Interruption of care can be life threatening and may have detrimental effects on the patients' physical and mental well‐being.^14^, ^35^

### 2.1 Deprivation of resources and access to healthcare services

During periods of epidemics one of the issues that arise is that there is an increased need for healthcare professionals to provide care to patients infected with the specific infectious agent. The latter may lead to deprivation of the necessary resources that are needed to treat patients with chronic disorders including cancer patients. For example, it has been estimated that since January 2020, more than 30 000 medical workers travelled from areas around Wuhan to Wuhan to help manage COVID‐19 patients and contain the outbreak. This phenomenon affected healthcare services outside Wuhan since there was a decrease in the number of doctors and other healthcare professionals in those regions.^23^ In addition, enforced quarantine, as was the case in Wuhan, caused complications in terms of attendance of cancer patients for scheduled appointments and continuity of care. There were also reported issues with severe complications or emergencies particularly in patients with advanced cancers due to their inability to access required healthcare.^27^

Similar to China, Italy has also been severely affected by the COVID‐19 epidemic. Hospitals of affected areas had to re‐allocate resources for the epidemic. Treatments for cancer patients were postponed so that medical teams could deal with the overwhelming number of COVID‐19 patients in the Intensive Care Units (ICUs).^36^ In Italy, specific protocols within the Emergency Medical System were applied to increase the capacity of Intensive Care Units.^37^, ^38^ In order to achieve this, in the most affected areas medical specialists, including oncologists, were asked to provide their assistance in managing patients suffering from COVID‐19 requiring hospitalization in ICUs or in the departments of infectious or respiratory diseases or general internal medicine.^39^ In addition to the organized re‐allocation of resources, in areas where COVID‐19 is widespread, physicians may be limiting in‐person contact with cancer patients due to concerns of being asymptomatic and exposing the patients to COVID‐19. In addition, physicians may limit exposure to patients because they may not have personal protection equipment (PPE) especially in areas and times when PPE is in shortage or is being redirected to COVID‐19 treating hospitals.

Access to healthcare services for cancer patients may also be limited because lockdown measures may have affected the social determinants of health.^36^ For example, in certain countries such as the USA where healthcare is provided by insurance, the processing or renewal of insurance paperwork may delay the onset or continuation of treatment. Furthermore, in parts of the world where transport is affected by lockdown (ie closing of roads and airports), patients may be restricted from travelling to other cities or countries for treatment to receive the necessary care. Moreover, it is important to bear in mind that in certain parts of the world, where patients have to pay for cancer services, lockdown may have affected their finances enough that they may either delay or may not be able to seek treatment.^40^

Currently, there are no official reports of how the treatment of cancer patients has been affected by the lack of resources and limited access to healthcare due to the COVID‐19 pandemic in most afflicted countries. Yet this is an important issue that should be carefully analysed and reported.

### 2.2 To treat or to defer?

No studies are yet available that have investigated the risk of untreated malignancy while waiting for COVID‐19 to subside vs the risk of exposure to the virus during cancer treatment.^14^ Usually the decision as to whether treatment should be offered or deferred is left to the judgment of multiprofessional healthcare teams and particularly oncologists who are responsible for the management of cancer patients. In making decisions regarding whether treatment should be offered as usual or deferred to reduce the risk of infection with COVID‐19, a number of factors should be considered since not all cancer patients fall under the same category or urgency for treatment (ie there are differences for each case with regards to the patient's age, type of cancer, stage of cancer, and the presence of other comorbidities). In addition, the risks vs benefits posed by the hospital setting need to be considered.^14^ Therefore, decisions with regards to patients' treatment are usually taken on a case by case basis which is time consuming further straining the limited resources available.

Some healthcare settings and guiding bodies seem to be in favor of deferring treatments they deem to be 'less urgent' for cancer patients.^41^ Some organizations such as the American College of Surgeons are giving guidance regarding Breast Cancer treatment based on the number of COVID cases in the area and the availability of hospital resources. For example, they are deferring surgeries in early stages of cancer.^34^ Researchers have also proposed delaying chemotherapy treatment and surgeries in stable cancer patients in places where COVID‐19 is endemic.^18^, ^41^ More specifically, Yu et al,^42^ suggested that if surgery is required for the management of colorectal cancer patients, it should be done as soon as the hospital can accept admissions and that surgeons should use laparoscopic surgery vs open surgery under these conditions.

In the UK, up until March 2020, even though England, Scotland, and Wales cancelled 'less urgent' surgeries, the policy was to continue to treat cancer patients in the same way as prior to the start of the COVID‐19 epidemic.^43^ In fact the UK government introduced specific guidelines for cancer patients on the 21st March strongly advising them to stay at home and avoid person to person contact.^44^ However, the latter recommendation could not be applied in the case of cancer patients on active treatment plans such as chemotherapy and radiotherapy which is offered in hospitals.^45^

Evidence in the literature supports that, for example in the case of colorectal cancer, the 3‐10 year survival is lower if treatment starts beyond 5 days from diagnosis^46^ and that the ideal time for colon resection is between 3‐6 weeks.^47^ Yet the latter is unlikely to be achieved for most colon cancer patients during the COVID‐19 pandemic and particularly in countries which are severely affected by the virus.^30^ A delay in treatment may have detrimental effects for the patients' prognosis and quality of life. In addition, a delay in the treatment of cancer is associated with an increased cost of care.^36^, ^48^ Therefore, by delaying the treatment of cancer, financial and healthcare resources are wasted which could have been used more effectively in developing a vaccine or treatments for COVID‐19 and/ or better treatments for colon or other types of cancer.

In a paper by Cortiula et al,^49^ the researchers expressed their concern regarding how re‐allocation of healthcare personnel to the COVID‐19 triage and management may leave uncovered some vital activities for cancer patients such as treatment and surgeries in Italy. They emphasized how delayed treatment may have detrimental effects for cancer patients since it may lead to disease progression particularly in patients in the most advanced stages of cancer. They proposed that patients with advanced disease, and no suggestive symptoms of COVID‐19, should continue to receive chemotherapy or radiotherapy treatment, without unnecessary delays. Cortiula et al,^49^ also mentioned that even screening appointments should be kept or re‐scheduled shortly after cancellation, whereas others proposed the need to postpone planned cancer screening procedures.^40^ Cortiula et al,^49^ also expressed concerns that during the pandemic it may not be possible to deliver palliative care to patients unable to move from their homes to sites of treatment due to the quarantine.

The Italian Association of Medical Oncology (AIOM) in collaboration with the Boards of Academic Oncologists (COMU) and the Oncology Unit Directors (CIPOMO) have recently proposed some important recommendations for cancer patients.^39^, ^50^, ^51^ For patients currently receiving active treatments, the recommendations propose that oncologists should consider the possibility of a delay in treatment on a case‐by‐case basis. The decision should be based on the cancer type and stage, the clinical condition of the patient, the treatment indicated for the condition, the patient's response to anticancer therapy, and the potential risks for an infection with SARS‐CoV‐2. For patients that have completed their treatment and are in the follow‐up phase, the recommendations propose that oncologists should avoid asking patients to come to the hospital for routine follow‐up visits. Instead they should try to use telephone calls or Telemedicine for patient consultations.^51^ Oncologists should limit consultations at the hospital to cases of patients who report new symptoms or signs of disease progression. Regarding admission to the hospital, outpatients scheduled for treatment should attempt to go alone and avoid the assistance of a caregiver except for cases when the latter is unavoidable. In addition, triage of patients with fever and/ or respiratory symptoms should be applied to prevent possible exposure to other patients and healthcare providers.^39^, ^50^, ^51^

In the USA, even though until March 2020 there was no suggested interruption of care for cancer patients, researchers started proposing the postpone treatment for some cancer patients. For example, Ganatra et al,^13^ proposed that for patients with stable cancer it may be advisable to consider postponing anti‐neoplastic therapy or 'less urgent' surgery in areas with high transmission of SARS‐CoV‐2. They emphasized that the decision should be made on a case‐by‐case basis after consideration of the overall health of the patient and the type of anti‐neoplastic therapy needed. Patients with a recent history of stem‐cell transplantation in areas of high transmission, should 'self‐quarantine' to avoid exposure to the virus. Also, carers of such high‐risk patients should also try to 'self‐quarantine' or obtain testing if they have any concerning symptoms. Ganatra et al,^13^ also proposed that in‐person visits could be substituted with telehealth visits and any nonurgent procedures should be deferred.

^58^Due to the increasing burden of COVID‐19 in the USA since March 2020,^52^ as of 4th May 2020 several guiding bodies have published guidance on different types of cancer. The American Society of Breast Surgeons has published brief, high level guidance on prioritization of care in breast cancer.^33^ The American College of Surgeons has published guidance on triage of patients with breast cancer for surgery.^34^ Overall, the guidance provides recommendations to delay cancer treatment including surgeries based on the type/risk of the cancer patient and the risk/burden to healthcare in the area.^33^, ^34^ In addition, an American multiorganizational panel has published recommendations on the triage, prioritization, and treatment of breast cancer ^53^ and an international group has also published recommendations on the same topic.^54^ Currently, cancer type specific guidance has been developed for a number of cancers including gastrointestinal cancer,^55^, ^56^ genitourinary cancers,^57^, ^58^, ^60^ gynecological cancers,^59^, ^60^ head and neck cancer,^61^, ^62^ hematological malignancy,^63^, ^64^ hepatocellular carcinoma,^65^ lung cancer,^66^ neuroncology,^67^ and skin cancer.^68^ The Society of Surgical Oncology has published a brief guidance on surgery which is specific for each type of cancer.^69^

### 2.3 Special measures before, during, or after surgeries?

Another matter to be addressed is whether special measures should be implemented before, during, and after surgeries for cancer patients. Wen and Li^70^ recommended full assessment of the patient for infection in order to detect asymptomatic cases, history of recent travel and contact with COVID‐19 infected people, body temperature measurement and chest CT scan. In addition, Li et al,^41^ recommended a 2‐week isolation of the patient and checking body temperature twice daily as well as testing the patient for the virus. Yu et al,^42^ proposed complete examinations of cancer patients for COVID‐19 before operation and using an operating room with a negative pressure system. In addition, they emphasized the importance of following the sterilization and disinfection protocols for surgical equipment and the use of general anesthesia with tracheal intubation. The authors also proposed that the protocols for handling infected specimens should be used for handling all surgical specimens.

### 2.4 Concerns with stem cell transplantations

Some cancer patients may have to undergo planned allogeneic stem cell transplantation which poses certain challenges for both the healthcare team and the patients. It has been recommended that stem cell transplantation could be reasonably delayed in view of COVID‐19, particularly in a situation where the disease is controlled with conventional treatment.^36^ In addition, visitors to post‐transplant patients should be restricted in order to reduce the risk of infection of the immunosuppressed patients with SARS‐CoV‐2.^71^

Another issue related to stem cell transplantations is the supply of stem cells for patients in need which have been reported due to travel restrictions. Stem cells need to be transplanted within 72 hours and the latter is risky given the new developments with travel restrictions.^49^ DKMS is a collection of charities working in seven countries around the world to recruit donors for people in need of blood stem‐cell transplants. Since the COVID‐19 outbreak DKMS started exploring the possibility of having the stem cells transported in cargo flights. In addition to ensuring that stem cells are available to patients at the right time, the outbreak itself has complicated the process of donations. The process requires donors to attend hospital for preparation and blood tests and the donation can take a couple of days and donors have concerns with regards to their safety. It is therefore expected that in the next few months the supply of stem cells may be affected dramatically, and actions should be taken immediately to address this matter which may have detrimental effects for a high number of cancer patients across the globe.^49^

### 2.5 Concerns regarding access to supportive services

In addition to the need for access to medical treatment (such as chemotherapy and hormonal therapy, radiotherapy and surgery), cancer patients and survivors need to have access to supportive services such as physical therapy and occupational therapy. Evidence in the literature suggests a significant role of physical therapy and occupational therapy in decreasing the symptom burden and improving the strength, endurance, and physical functioning of cancer patients^72^ Therefore, supportive care involving physiotherapy and occupational therapy ameliorates the multiple physical and psychosocial challenges faced by many cancer patients and survivors.^72^, ^73^ Due to the fact that these therapies improve the cancer patients' and survivors' quality of life, it is important to consider how access to such services may have been affected during the COVID‐19 pandemic. It is well‐known that in‐person appointments of occupational therapy and physical therapy have been interrupted or significantly reduced in most countries. However, some national organizations have provided recommendations with regards to the provision of physical therapy and occupational therapy during the COVID‐19 pandemic. For example, CDC has asked physical therapists to assess if each of their patients would be a good candidate for physical therapy via telehealth/telemedicine, if a patient's therapy could be delayed, or if the patient would be a good candidate for physical therapy in the home.^74^, ^75^ Despite the lack of availability of published data on this matter, future publications may provide evidence on the impact of the interruption of physical and occupational therapy, with or without the development of alternative ways of therapy, during the COVID‐19 on the cancer patients' quality of life.

### 2.6 Are there any specific challenges for pediatric cancer patients?

It is generally considered that children are less likely to develop severe COVID‐19 illness compared to adults. Nevertheless, a study has highlighted that infants and younger children (ie ≤ 5 years) are more likely to develop severe clinical manifestations compared to older children (ie ≥ 6 years) possibly due to the immaturity of the immune system of the former.^76^, ^77^ One problem with pediatric cancers is that they are usually aggressive and need intensive treatment with a combination of chemotherapeutic agents which induce severe immunosuppression to these patients.^76^ Therefore usually there is no option to delay treatment of pediatric cancers as suggested for other types of cancers.^18^ Even though some patients are hospitalized and are in isolation, many children are treated as outpatients. The latter places a certain risk for these patients since during their treatment appointments they may be exposed to SARS‐CoV‐2. The latter has caused anxiety in young cancer patients and their families and this has led to the development of specific recommendations by national authorities to prevent the spread of SARS‐CoV‐2 in this vulnerable population.^76^ These recommendations are of major significance since they provide a reference to healthcare teams and caregivers.^50^ Through these recommendations, efforts have been made to protect pediatric cancer patients by minimizing the number of patients visiting the oncology clinics, reducing visits, and using telehealth when possible.^76^

### 2.7 Additional concerns

In addition to the usual questions with regards to the delivery of care to cancer patients, oncologists are being called to look into the specific biology of the cancer and address specific concerns. For example, many patients undergoing cardiotoxic chemotherapy may be on an angiotensin‐converting enzyme inhibitor (ACEi) or angiotensin receptor blocker (ARB). Whether treatment with an ACEi or ARB increases the risk associated with COVID‐19 is controversial but should be investigated.^78^, ^79^ In addition, COVID‐19 seems to predispose patients to thrombotic disease, both in the venous and arterial circulations.^80^ This is particularly important in cancer patients who are at higher risk of thrombotic events and some of whom may have a history of thrombotic events. There is no clarity yet over whether any medication should be used for the prophylaxis of cancer patients or for the management of cancer patients infected with COVID‐19.^80^ Last but not least, it is important to consider any possible shortages in cancer medication. In the UK no shortages of cancer medication were reported.^43^ As time progresses, it is likely that there will be effects in terms of the manufacture, supply, and shipping of drugs used in cancer treatment.^76^

## 3 SAFETY OF CURRENT THERAPIES USED FOR THE MANAGEMENT OF COVID‐19 IN CANCER PATIENTS

Currently, there is neither a vaccine nor a direct acting antiviral that can be used for the management of COVID‐19. Therefore, there is an urgent need to develop both a vaccine and drugs to manage COVID‐19. It is believed that the first vaccine will be available 18 months after the outbreak.^81^ Currently, the disease is managed by symptomatic therapy and the Intensive Care Unit is used for the management of patients with severe disease and those that have organ involvement or require intubation.^82^

Currently there are more than 80 clinical trials in an effort to find the most appropriate coronavirus treatment.^83^ Some of the drugs currently used for the management of COVID‐19 include Chloroquine,^83^ Remdesivir (GS‐5734),^84^, ^85^ Lopinavir/Ritonavir,^86^, ^87^, ^88^ and Favipiravir.^84^ Given that clinical trials are at the early stages, it is difficult to assess if the drugs are safe to use in cancer patients or whether there are any contra‐indications for these group of patients based on their immunosuppression or other ongoing treatments. As the results of clinical trials are analysed it will be important to evaluate any possible safety issues and side effects imposed by these medications on cancer patients. Drug‐drug interactions remains a possibility which has not been fully investigated yet.

Currently, there is no evidence regarding the use of prophylactic antiviral therapy for COVID‐19 in immunosuppressed patients such as cancer patients. In the ongoing clinical trials the treatments have been used in patients with confirmed infection and not for prophylactic use.^76^ Therefore, at the current time there is no recommendation to offer any of these pharmaceutical agents in immunocompromised patients, including cancer patients, to protect them from getting infected with COVID‐19. This, however, could be a possibility in the future since there is an additional need to protect this group of patients due to their immmunompromised state.

In a recent study by Henry et al,^89^ the researchers reported on a cohort of 2500 French cancer patients who were treated with different drugs and methylene blue. During the COVID‐19 epidemic, none of the patients developed influenza‐like illness. While this lack of infection could be by chance, it is possible that methylene blue had a preventive effect for COVID‐19 infection in this group of patients. This is in line with the antiviral activity of Chloroquine, which is a Methylene blue derivative. This is another area that could be further studied in the future in larger randomized control trials in order to clarify if indeed such an agent could be safely used in cancer patients.^89^

## 4 PSYCHOEDUCATION AND EMOTIONAL SUPPORT FOR CANCER PATIENTS DURING THE COVID‐19 PANDEMIC

Based on the increased vulnerability of cancer patients, special efforts should be put in place to educate cancer patients with regards to their personal protection. The WHO and Centers for Disease Control (CDC) have advised that every individual should frequently wash their hands, avoid touching their face, and practice social distancing.^4^, ^52^ While these are important general guidelines for the entire population, cancer patients should strictly follow these guidelines given that they may be more likely to acquire the infection and develop severe disease.^14^ However, there are no specific guidelines regarding the use of face masks in cancer patients and this should be explored.^76^

It is likely that the COVID‐19 pandemic causes increased anxiety to cancer patients with regards to their risk, what they can do to protect themselves and how their treatment may be affected. Therefore, psychoeducation of cancer patients via a variety of different mechanisms is of critical importance at this time, and cancer treatment centers, in collaboration with cancer patient societies, should do their best to offer advice and support for cancer patients. Virtual informational sessions, telephone support lines, social media, and webpages could do a lot in providing a forum where each patient can express their concerns and receive detailed and up to date information with regards to how COVID‐19 affects their treatment. The Seattle Cancer Care Alliance in collaboration with the Fred Hutchinson Cancer Research Center, and the University of Washington have developed patient handouts and a website to educate and support cancer patients and their family on how to prevent infection with SARS‐CoV‐2. In addition, they have developed a telephone triage line for patients with mild symptoms in the community to minimize exposures at the clinic. Testing at a drive‐up site was also coordinated when appropriate.^90^ This provides an example of how healthcare organizations, together with research institutes and patient groups, can work together to provide better education and thereby prevent cancer patients from getting infected with SARS‐CoV‐2.

There is no doubt that in addition to the effects of COVID‐19 on people's physical health, the virus has affected the emotional status of people around the world.^91^ Patients suffering from chronic disorders, such as cancer patients, are extremely vulnerable since they are commonly psychologically strained and the emotional aspect of their disorder may not be sufficiently managed as part of their care. A study by Zheng et al,^92^ assessed depression in cancer patients during the COVID‐19 epidemic. A survey was conducted in patients of Zhejiang Cancer Hospital through social media platforms. The Self‐Rating Depression Scale (SDS) was used to evaluate the effect of COVID‐19 on cancer patients. In this study, the incidence of depression in cancer patients during the coronavirus pandemic was found to be 40.7% (71.4% had mild depression and 28.6% had moderate depression). The COVID‐19 pandemic had an impact on mood, sleep, and stress in 19.8%, 5.8%, and 17.4% of the cancer patients respectively. While few studies have been carried out thus far to investigate the effect of the pandemic on cancer patients, preliminary evidence suggests that psychological support should be provided to these patients. Despite the common constraints of physical interactions, consultations could be replaced by virtual or telephone consultations to ensure the holistic continuity of care for this group of patients during a difficult period in their lives which imposes another level of stress to their previous strained psychology.^35^, ^93^ In addition, further research is needed to determine the best way to utilize virtual platforms to effectively conduct such psychological individual or group support.

## 5 CLINICAL TRIALS WITH CANCER PATIENTS

COVID‐19 is hindering research in all disciplines, including cancer research. Oncology trials are resource‐intensive, and approved protocols frequently require in‐person contact between participants, researchers, and the medical team.^35^ CDC has provided recommendations for cancer patients participating in clinical trials. They have proposed screening patients for fever/symptoms, postponing nonessential in‐person visits, and arranging for telemedicine interactions when possible. In addition, they are proposing that efforts should be made to provide personal protective equipment with the aim to protect both the immunocompromised participants and also the medical and research teams.^35^

The National Institutes of Health has recently released several helpful notices regarding funded human subject research trials during the COVID‐19 pandemic in the USA. These include encouraging investigators to consider limiting study visits to those needed and to conduct virtual visits when possible.^94^ In addition, it is very important to be respectful of the participants' right to withdraw from a clinical trial if they feel that their participation in the trial poses an increased risk to their health.^35^

In the UK, Trusts are making decisions around clinical trials by following government advice and on a case‐by‐case basis. In certain cases, this prevented the start of new trials or the recruitment of existing trials, but attempts were made to continue existing clinical trials. For example in certain cases there was delivery of medication to people's homes to avoid increased traffic at hospitals.^43^ The European Commission, the European Medicines Agency (EMA) and the Head of Medicines Agency (HMA) as well as the Food and Drug Administration, and the Italian Medicines Agency have issued special guidance for the conduction of clinical trials during the COVID‐19 emergency.^95^, ^96^ The guidance includes a harmonized set of recommendations to ensure the safety of the trial while preserving the quality of the data generated by the trials. The guidance provides information on changes and protocol deviations which may be needed in the conduct of clinical trials to deal with extraordinary situations, eg if trial participants need to be in self‐isolation or quarantine, access to hospitals is limited due to the risk of spreading infections, and healthcare professionals are being reallocated.^95^, ^96^

## 6 CONCLUSIONS

The experiences of the COVID‐19 pandemic have shown that the healthcare systems in most countries around the globe were not ready to deal with the effects of the pandemic. In addition, while efforts are still focused on the management of COVID‐19 infected patients, it is important to also address the needs of patients suffering from chronic disorders such as cancer patients who may be affected either directly or indirectly by COVID‐19.

Multiprofessional heathcare teams address the needs of different cancer patients (ie from patients on active treatment to patients on a follow‐up phase). Cancer is a very heterogeneous disease which is why oncologists routinely review each patient individually by taking into consideration a number of different factors, such as the stage of the disease, type of cancer, clinical outcome, and response of the patient.

During the COVID‐19 pandemic, there have been some attempts for the development of recommendations for the management of cancer patients during this extremely difficult period.^35^, ^40^ It is important that more specific guidelines are developed as a collaboration between infectious disease doctors, epidemiologists, oncologists as well as other members of the multiprofessional heathcare teams who are directly involved with the care of cancer patients. The guidelines should be specific to particular types and stages of cancer (from diagnosis to active treatment to palliative care to survivorship) and to particular treatments (eg chemotherapy, radiotherapy, surgery) and there should be specific guidelines for pediatric patients.

In conjunction with guidelines, the details of each individual patient should still be reviewed, and a final management plan should be developed for each patient. Once the management plan is developed, it should be clearly communicated to the cancer patients. Physical consultations could be replaced by telephone or Telemedicine consultations whenever it is considered safe to do so. The patients should receive clear explanations with regards to their management but also with regards to their personal protection and safe practices. Further research should be conducted on the best way to ensure optimal and effective care of cancer patients using virtual platforms. In addition, although the evidence may not be available yet, in a few months data will be available that should be analysed to evaluate the impact of COVID‐19 on cancer patients. The results of such analyses are necessary to contribute toward the development of evidence‐based guidelines for the management of cancer during the COVID‐19 pandemic.

In addition to developing guidelines for the management of cancer patients during COVID‐19, the most promising medication(s) that will eventually be used for the management of COVID‐19 will need to be evaluated in cancer patients. In addition, more specific guidelines will need to be developed on the conduct of clinical trials, and the possibility of future pandemics should be included in the design of future clinical trials. In addition, psychological support should be continued to be provided to cancer patients and adapted to address their increased anxiety during the COVID‐19 pandemic.

In summary, the COVID‐19 epidemic has revealed that the healthcare systems have difficulties not only in dealing with the direct effects of the epidemic but are also faced with challenges and dilemmas with regards to the continuity of provision of healthcare to cancer patients. The epidemic has revealed the need to develop a holistic contingency plan for the management of cancer patients. A holistic contingency plan is both urgent and vital to minimize the increased morbidity and mortality that could be caused by the deprivation of high‐quality healthcare to cancer patients during the current or future pandemics.

## Conflict of Interest

The authors declare no conflict of interest.

## Authors Contribution

Constantina Constantinou designed and wrote the manuscript. Ourania Kolokotroni, Maria‐Cecilia Mosquera, Alexandros Heraclides, Christiana Demetriou, Peter Karayiannis, Annalisa Quattrocchi, and Andreas Charalambous contributed to the review of individual sections and the overall revision of the manuscript. All authors reviewed and approved the final version of the manuscript.

## Data Availability

This is a review article and therefore the data discussed in this study are publicly available.
